# Association between RCAS1 expression and clinical outcome in uterine endometrial cancer

**DOI:** 10.1038/sj.bjc.6601126

**Published:** 2003-07-29

**Authors:** K Sonoda, S Miyamoto, T Hirakawa, T Kaku, M Nakashima, T Watanabe, K Akazawa, T Fujita, H Nakano

**Affiliations:** 1 Department of Obstetrics and Gynecology, Graduate School of Medical Sciences, Kyushu University, Maidashi 3-1-1, Higashi-ku, Fukuoka 812-8582, Japan; 2 School of Health Sciences, Kyushu University, Maidashi 3-1-1, Higashi-ku, Fukuoka 812-8582, Japan; 3 Department of Molecular Immunology, Medical Institute of Bioregulation, Kyushu University, Maidashi 3-1-1, Higashi-ku, Fukuoka 812-8582, Japan; 4 Department of Medical Informatics, Niigata University Medical Hospital, Niigata University, Asahimachi-dori 1-754, Niigata 951-8520, Japan

**Keywords:** RCAS1, uterine endometrial cancer, immunohistochemistry, prognostic factors

## Abstract

RCAS1, which acts as a ligand for a putative receptor on immune cells such as peripheral lymphocytes and natural killer cells, is strongly expressed in human cancers. RCAS1 can induce these cells to undergo apoptotic cell death, which suggests that RCAS1 expression may prohibit the stromal reaction occurring in a tumour. To clarify the clinical significance of RCAS1 expression in uterine endometrial cancer, we analysed the association between RCAS1 expression and clinicopathologic variables by statistical methods. With the use of immunohistochemical techniques, we performed a retrospective study of RCAS1 expression in resected tumour tissue from 147 patients with uterine endometrial cancer. We evaluated the statistical correlation between RCAS1 expression and clinicopathologic variables. RCAS1 was expressed in 106 of 147 patients with uterine endometrial cancer ; 30 of these 147 patients showed RCAS1 overexpression. Overexpression of RCAS1 was significantly correlated with age at surgery, stage, extent of myometrial invasion, and positive peritoneal cytologic results. Multivariate analysis revealed that RCAS1 expression and metastasis were clinically significant prognostic factors for the overall survival. These findings indicated that analysis for RCAS1 expression can provide crucial information about the clinical behaviour of uterine endometrial cancer, which may be valuable for the management of patients with this disease.

Uterine endometrial cancer is one of the most frequently diagnosed gynaecologic malignancies in Western countries. Approximately 38 300 new cases and 6600 deaths caused by uterine endometrial cancer occurred in the year 2001 in the US (American Cancer Society, 2002). In Japan, the incidence of uterine endometrial cancer has been reported as 22% of all uterine malignancies, with 1133 deaths caused by this disease in 2001 (Japan Society of Obstetrics and Gynecology, 1998; National Cancer Center, 2002). The best prophylaxis for uterine endometrial cancer and the precursors of this disease is thought to be the early detection. The risk of recurrence is directly related to surgical stage; extension of tumour cell invasion into the cervix; positive peritoneal cytologic results; metastases including involvement of lymph nodes, adnexa, and other organs; degree of myometrial invasion; and tumour differentiation (DiSaia and Creasman, 1997). Survival of patients with uterine endometrial cancer thus depends on these clinicopathologic factors.

Alterations in the transforming genes including K*-ras* and c-*erb*B2/*neu* oncogenes and the *p53* and *PTEN* tumour suppressor genes are associated with the development of uterine endometrial cancer (Enomoto *et al*, 1990; Kohler *et al*, 1992; Bancher-Todesca *et al*, 1998; Maxwell *et al*, 1998; Esteller *et al*, 1999). Of various molecular markers, the following have been reported as possible prognostic factors for this disease: angiogenetic factors, epidermal growth factor receptor (EGFR), hormonal receptors including Oestrogen receptor and progesterone receptor, and motility-related protein 1 (MRP1)/CD9 (Chambers *et al*, 1988; Ingram *et al*, 1989; Morgan *et al*, 1996; Ebert *et al*, 2000; Miyamoto *et al*, 2001; Sivridis, 2001). Although several molecular alterations in uterine endometrial cancer have been identified, few reports have demonstrated the significant molecules in a large number of clinical samples. To improve the clinical outcome for this disease, these crucial molecules should be identified so as to allow their use as clinical prognostic factors.

RCAS1 (receptor-binding cancer antigen expressed on SiSo cells) is a type II membrane protein able to form oligomers through a coiled-coil structure in its C-terminal portion (Nakashima *et al*, 1999). RCAS1 acts as a ligand for a putative receptor present in various human cell lines and on normal peripheral lymphocytes. RCAS1 inhibits the *in vitro* growth of receptor-expressing cells and induces apoptotic cell death. RCAS1 was strongly expressed in uterine and ovarian malignancies (Sonoda *et al*, 1996; Razvi *et al*, 1999). In different human cancers, RCAS1 expression has been associated with clinical outcome. RCAS1 expression was significantly related to the overall survival of patients with uterine cervical adenocarcinoma or non-small-cell lung carcinoma (Kaku *et al*, 1999; Iwasaki *et al*, 2000; Izumi *et al*, 2001). RCAS1 expression has also been reported to correlate with tumour progression or the invasive tendency of uterine cervical, gastric, skin, and hepatocellular carcinomas (Sonoda *et al*, 1998; Kubokawa *et al*, 2001; Noguchi *et al*, 2001; Takahashi *et al*, 2001). Thus, RCAS1 may play a pivotal role in the aggressive behaviour of a tumour in humans. We previously reported that RCAS1 expression increased during carcinogenesis of uterine endometrial cancer (Sonoda *et al*, 2000). To clarify the clinical prognostic significance of RCAS1 expression in uterine endometrial cancer, we analysed the univariate and multivariate association between RCAS1 expression and clinicopathologic variables in 147 patients with this disease.

## MATERIALS AND METHODS

### Patients and surgical specimens

All the patients with uterine endometrial cancer in this study had undergone surgery between January 1979 and February 1999 at the Department of Obstetrics and Gynecology, Kyushu University Hospital. The mean age of all patients was 54.3 years, with a range of 27–82 years. The mean duration of follow-up for all patients was 195.7 months, with a range of 4–618 months. The histologic subtype of all cases was endometrioid adenocarcinoma. Clinical staging of the 147 cases was as follows: 77, 25, 35, and 10 cases were classified as stages I, II, III, and IV, respectively. The specimens in this study were graded according to the 1988 International Federation of Gynecology and Obstetrics criteria. Slides of both curettage specimens of uterine endometrium and hysterectomy specimens were available for this study. All specimens were fixed, embedded in paraffin, and stained with haematoxylin and eosin. Histologic methods were used for evaluation of the slides of the specimens to determine the grade of the carcinoma, depth of myometrial invasion, cervical invasion, invasion of the lymph–vascular space, and metastases of lymph nodes and other organs. With regard to metastases, 22 cases had lymph node metastases, 12 cases had adnexal metastases, four cases had rectal metastases, one case had bone metastases, and two cases had multiple metastases. A peritoneal washing was also evaluated by cytologic methods.

In this study, 50 patients were treated by surgery alone (total abdominal hysterectomy, bilateral salpingo-oophorectomy, pelvic and para-aortic lymphadenectomy). After the operation, certain patients received additional therapy: 39, 15, and 14 cases received radiation, hormonal therapy, and platinum-based chemotherapy, respectively; and 29 cases received a combination of surgery, radiation, and hormonal treatment or platinum-based chemotherapy. The overall survival time was defined as the time from the surgery date to the final date of observation in this study (31 March 2002) or the time from the surgery date to the date at death caused by the cancer.

Tissue specimens of uterine endometrial hyperplasia were obtained from 30 patients at surgery. These 30 endometrial hyperplasia samples included 10 cases each of simple, complex, and atypical hyperplasias. Normal uterine endometrial tissue specimens were also obtained at surgery from 30 Japanese patients: 10 cases in the follicular phase of the menstrual cycle, 10 cases in the secretory phase of the menstrual cycle, and 10 cases in the postmenopausal state.

Informed consent was obtained from all patients.

### Immunohistochemistry

For immunohistochemical analyses, one or two representative sections were selected for each case, and the streptavidin–biotin method was used for formalin-fixed, paraffin-embedded specimens (Hsu *et al*, 1981). Sections 4-*μ*m thick were cut from paraffin-embedded blocks. Endogenous peroxidase was blocked with 3% hydrogen peroxidase for 5 min. The slides were then washed twice in Tris-buffered saline (TBS) and were incubated with normal goat serum diluted in a cell staining buffer (TBS containing 0.1% bovine serum albumin and 0.01% sodium azide) for 30 min. Next, mouse anti-human RCAS1 monoclonal antibody was applied, and the slides were incubated in a moist chamber for 30 min. After two additional washes, the sections were incubated for 30 min with biotinylated second antibody (goat anti-mouse immunoglobulins; DAKO, Glostrup, Denmark). The sections were washed three times in TBS and were incubated with avidin-biotinylated peroxidase complex (Strept ABC complex/horseradish peroxidase; DAKO) for 30 min. After three additional washes in TBS, a 3,3′-diaminobenzine tetrahydrochloride working solution was applied. The sections were then counterstained in haematoxylin and mounted in Permount. The entire procedure was performed at room temperature. Negative controls were treated in the same way, but anti-RCAS1 monoclonal antibody was replaced by mouse IgM.

The 30 specimens of normal endometrium and 34 specimens of endometrial cancer were also stained with anti-TNF-*α* (Santa Cruz Biotechnology, Santa Cruz, CA, USA) and anti-Fas-L (Histofine, Nichirei, Japan) monoclonal antibodies, and the relation between the expression of RCAS1 and that of TNF-*α* or Fas-L was evaluated.

### Specimens classified on the basis of immunohistochemical results

The immunohistochemical expression of RCAS1, TNF-*α*, and Fas-L was reviewed independently by two observers (K Sonoda and S Miyamoto) who had no knowledge of the clinicopathologic data. Five representative fields were examined, and a total of 1000 tumour cells (200 for each field) was counted via a microscope with a high-power field (× 400) objective. The observers counted each slide twice, so that there were four counts for each slide, and the average of the four scores was recorded. Criteria for scoring the overall extent of immunoreaction were as follows: more than 50% positive cells=overexpression, from 25 to 50% positive cells=positive expression, and less than 25% positive cells=normal expression.

For evaluation of TNF-*α* and Fas-L expression, we examined 30 specimens of normal endometrium and 34 specimens of endometrial cancer. The latter consisted of 12 cases with normal expression of RCAS1, five cases with positive expression of RCAS1, and 17 cases with overexpression of RCAS1. The numbers of cells positive for TNF-*α* and Fas-L expression among the 1000 tumour cells in the tissue sections were counted.

### Statistical analysis

The Fisher exact test, for each clinicopathologic factor and the expression levels of TNF-*α* and Fas-L, was used to find the significant factors that affected the expression of RCAS1 as univariate variables. The Mann–Whitney test was performed to check the equality of the distribution of age at surgery between the group showing RCAS1 overexpression and the other groups of RCAS1. The Overall survival curves were estimated by using Kaplan–Meier methods and were analysed by the log-rank test. Cox's proportional hazards regression analysis for the overall survival was used to select a set of prognostic factors from the nine variables, which were RCAS1 plus eight factors given in the first column of Table 1

**Table 1:** Relation between RCAS1 expression and clinicopathologic data

	**RCAS1 expression^a^**		
**Clinicopathologic variables**	**Others (*n*=117)**	**Overexpression (*n*=30)**	***P*-value**
Age (years; mean±s.d.)	52.7±9.6	60.8±9.2	<0.0001
*Surgical stage*			0.009
I	69	8	
II	16	9	
III	26	9	
IV	6	4	
			
*Histologic grade*			0.271
G1	77	15	
G2	20	8	
G3	20	7	
			
*Myometrial invasion* (%)			0.043
<50	78	14	
>50	39	16	
			
*Cervical stromal invasion*			
Negative	115	30	0.470
Positive	2	0	
			
*Lymph – vascular space invasion*			0.097
Negative	88	18	
Positive	29	12	
			
*Metastases*^b^			0.456
Negative	86	20	
Positive	31	10	
			
*Peritoneal cytologic results*			0.017
Negative	100	20	
Positive	17	10	

a Criteria for scoring RCAS1 expression were as follows: overexpression=more than 50% RCAS1-positive cells; others=from 0 to 50% RCAS1-positive cells.

b Metastases included involvement of lymph nodes and adnexa and metastases in other organs.

. Likelihood ratio tests, with a significance level of 0.05, were used to enter or remove factors at each step in the forward stepwise method. Statistical analysis was performed with the BMDP 3D, 2L computer package (BMDP, Los Angeles, CA, USA) and Statxact (Cytel Software Co., Cambridge, MA, USA).

## RESULTS

### Immunohistochemical detection of RCAS1 in endometrial cancer

**Figure 1:**
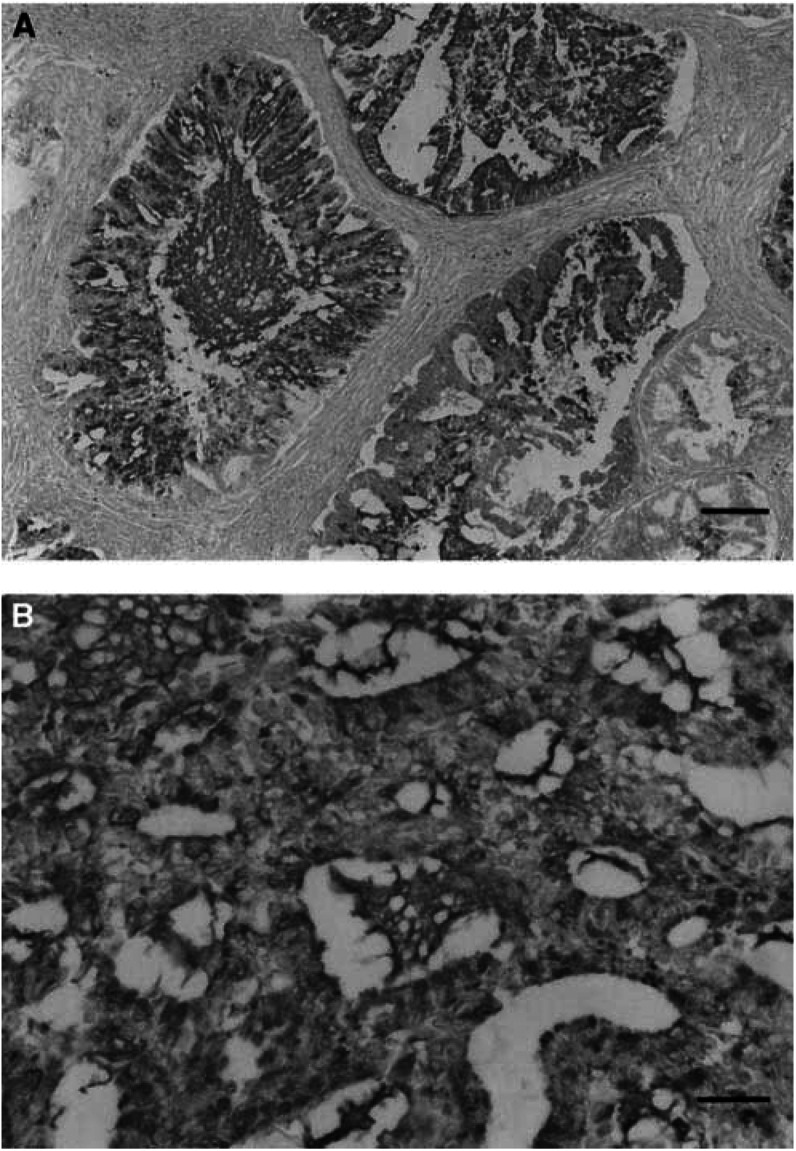
Immunohistochemical staining for RCAS1. This specimen showed RCAS1 overexpression. RCAS1 existed both in the cytoplasm and on the cell membrane of uterine endometrial adenocarcinoma cells. RCAS1 was also detected in the glandular lumen of the tumour cells (counterstained with haematoxylin; original magnification, (**A**) × 100, bar 50 *μ*m; (**B**) × 200, bar 25 *μ*m).

Diffuse staining for RCAS1 was observed both in the cytoplasm and on the cell membrane of cancer cells (Figure 1

). RCAS1 expression was detected in eight of 30 cases with normal uterine endometrium. Seven of these eight cases showed positive staining in 5–25% of cells; only one case showed positive staining in 25–50% of cells. In specimens of uterine endometrial hyperplasia, RCAS1 was expressed in less than 25% of cells in eight of 30 patients; positive RCAS1 staining in 25–50% of cells was found in three patients. The remaining 19 patients with endometrial hyperplasia demonstrated no expression of RCAS1. Among the 147 patients with uterine endometrial cancer, 51, 25, and 30 cases showed positive RCAS1 expression in 5–25, 26–50, and 51–100% of cells, respectively.

### Association of RCAS1 expression level with overall survival

**Figure 2:**
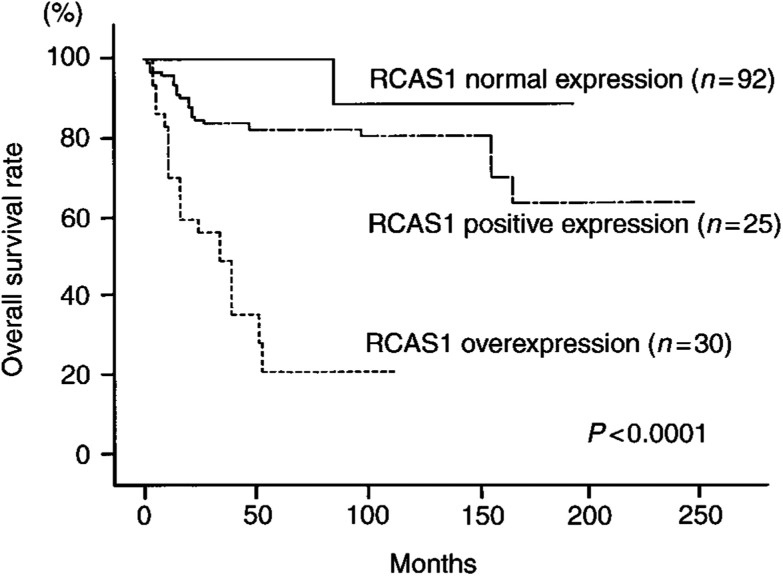
Overall survival of 147 patients with endometrial adenocarcinoma as related to RCAS1 expression (Kaplan–Meier estimates). The *P*-value was determined with the log-rank test. Patients with RCAS1 overexpression had a poorer survival than those with normal RCAS1 expression and those with positive RCAS1 expression (*P*<0.0001).

The overall survival curves according to the level of RCAS1 expression are shown in Figure 2

for 147 patients with endometrial adenocarcinoma. Patients with overexpression of RCAS1 had a significantly less favourable prognosis than those with normal expression and those with positive expression of RCAS1. The overall survival rates in the groups with normal expression, positive expression, and overexpression of RCAS1 were 96, 78, and 40%, respectively. The clinical outcome in patients with overexpression of RCAS1 was regarded as significantly poor (*P*<0.0001).

### Relation between immunodetected RCAS1 and clinicopathologic data

To evaluate the clinical significance of overexpression of RCAS1, we used statistical methods to analyse the relation between RCAS1 expression level and clinicopathologic variables. As shown in Table 1, the Fisher exact test for the group showing RCAS1 overexpression and the group showing the other RCAS1 expression levels revealed no statistically significant relation for the histologic grade, degree of cervical stromal invasion, degree of lymph–vascular space invasion, and metastases in patients with uterine endometrial cancer. Using the Mann–Whitney test, we found a significant difference in age at surgery between these two groups (*P*<0.0001). There were significant associations between overexpression of RCAS1 and surgical stage (*P*=0.009), extent of myometrial invasion (*P*=0.043), and peritoneal cytologic results (*P*=0.017).

### Prognostic value of RCAS1 expression level

Table 2

**Table 2:** Results of Cox's proportional hazards regression analysis

**Prognostic factor**	**Relative risk**	**95% confidence interval**	***P*-value**
RCAS1 (0: reduced, 1: overexpression)	8.191	3.990 – 16.816	<0.0001
Metastasis (0: negative, 1: positive)	4.464	2.280 – 8.743	<0.0001
Cervical stromal invasion (0: negative, 1: positive)	2.542	1.331 – 4.854	0.0047

shows the results of Cox's proportional hazards regression analysis performed with a stepwise method. Multivariate analysis suggested that overexpression of RCAS1 and metastasis were significant prognostic factors for the overall survival in patients with uterine endometrial cancer. However, only two of 147 patients with endometrial cancer had cervical stromal invasion, and they were alive at the final date of observation in this study (31 March 2002). Therefore, cervical stromal invasion was regarded as a clinically insignificant factor because of the small number of cases.

### Relation between RCAS1 and TNF-*α* or Fas-L expression level

In cases with normal endometrium, the numbers of cells positive for TNF-*α* and Fas-L expression were 32.2±11. 2 and 22.6±12.3 (mean±s.d.), respectively. All cases showed less than 10% expression of TNF-*α* and Fas-L. In cancer patients with normal expression, positive expression, and overexpression of RCAS1, the numbers of cells positive for TNF-*α* expression were 35.2±12.2, 29.2±12.5, and 30.4±14.2, respectively. The corresponding numbers for Fas-L expression in these groups were 21.2±10.3, 25.2±11.5, and 20.6±14.2. All these patients also showed less than 10% expression of TNF-*α* and Fas-L. These results indicated that RCAS1 expression had no association with TNF-*α* and Fas-L expression levels.

## DISCUSSION

In this study, a significant association was found between RCAS1 expression level and surgical stage (*P*=0.009), extent of myometrial invasion (*P*=0.043), and peritoneal cytologic results (*P*=0.017). These findings suggested that RCAS1 may have a role in the invasive properties of endometrial cancer. Patients with overexpression of RCAS1 had a significantly poorer prognosis than those with normal RCAS1 expression and those with positive RCAS1 expression. Multivariate analysis indicated that overexpression of RCAS1 and metastasis were independent significant prognostic factors for overall survival in patients with uterine endometrial cancer.

Fas-L-expressing carcinomas induce apoptosis in lymphocytes with Fas expression (O'Connell *et al*, 1996; Gastman *et al*, 1999). The inverse correlation between Fas-L expression and the number of CD8-positive tumour-infiltrating lymphocytes are reported in several malignancies (Ohno *et al*, 2000; Zietz *et al*, 2001). It has been thought that cancer cells expressing Fas-L have an advantage to evade antitumour immune surveillance in the course of an apoptotic tumour counterattack mechanism. In cultured cancer cells that express RCAS1, RCAS1 is secreted into the medium (Sonoda *et al*, 1996) RCAS1 is cleaved proteolytically by 12-*O*-tetradecanoylphorbol 13-acetate (TPA) as TNF-*α* and Fas-L are processed (manuscript in preparation). It is plausible that RCAS1 is secreted from the cancer cells with overexpression of RCAS1. RCAS1 induces apoptosis of lymphocytes by binding to a putative RCAS1 receptor (Nakashima *et al*, 1999). In addition, RCAS1 expression is associated with the number of apoptotic lymphocytes adjacent tumour cells in lung cancer and Hodgkin's disease (Iwasaki *et al*, 2000; Ohshima *et al*, 2001), and RCAS1 expression is inversely related with the degree of intratumoral infiltration of lymphocytes (Suzuki *et al*, 2001). In this study, TNF-*α* and Fas-L were little expressed in endometrial cancers. According to these evidences, lymphocyte apoptosis is possibly induced by the expression of RCAS1 in stromal tissue surrounding cancer cells with overexpression of RCAS1. Thus, RCAS1 may facilitate the invasion of cancer cells into connective tissue in endometrial cancer, because of an inhibition of the stromal reaction occurring in a tumour.

Reportedly, RCAS1 is localised to chromosome 8q23, and its expression is induced by oestrogen (Ikeda *et al*, 2000). Oestrogen receptor is expressed in both normal and hyperplastic uterine endometrial tissues (Lecce *et al*, 2001). In this study, RCAS1 was detected in less than 25% of cells in both of these uterine endometrial tissues. Therefore, RCAS1 expression in normal and hyperplastic uterine endometrial tissues may be mediated by activation of the Oestrogen receptor. However, a loss of Oestrogen receptor and progesterone receptor is associated with a clinically poor outcome (Niwa *et al*, 1999; Kounelis *et al*, 2000; Sivridis *et al*, 2001). Mediation by stimulation of Oestrogen production cannot be the primary explanation for the excess of RCAS1 in uterine endometrial cancer.

In principle, apoptotic factors including TNF-*α* and Fas-L are secreted by proteolytic processing and induce programmed cell death of target cells (Nagata, 1997; Baud and Karin, 2001). RCAS1 is also cleaved proteolytically. The ectodomain shedding of these factors is induced by addition of peptide growth factors and activation of mitogen-activated protein kinase (Fan and Derynck, 1999; Nath *et al*, 2001; Umata *et al*, 2001). In addition, expression of EGFR and HER-2/neu is associated with the aggressiveness of an uterine endometrial tumour, and expression of PCNA and Ki67 is correlated with clinical outcome (Khalifa *et al*, 1994; Niikura *et al*, 1995; Nordstrom *et al*, 1996; Fujiwaki *et al*, 1999; Rolitsky *et al*, 1999). According to these previous studies, the activation of mitogenic signals may be involved in the aggressive behaviour of uterine endometrial cancer. In cancer cells with aggressive potential, therefore, the excess of RCAS1 may have a role in the accelerated turnover of RCAS1 through ectodomain shedding. However, the molecular mechanisms for RCAS1 expression in aggressive endometrial cancer have remained obscure.

Our results presented here are the first to demonstrate that analysis of expression levels of RCAS1 can provide clinical information related to the aggressive behaviour of uterine endometrial cancer. Thus, evaluation of not only clinicopathologic parameters but also RCAS1 expression level may have clinical value for management of endometrial cancer patients.

In previous studies, RCAS1 expression was associated with poorer clinical prognosis for uterine cervical adenocarcinoma and non-small-cell lung carcinoma (Kaku *et al*, 1999; Iwasaki *et al*, 2000; Izumi *et al*, 2001). RCAS1 expression has also been reported to correlate with tumour progression or the invasive tendency of uterine cervical, gastric, skin, and hepatocellular carcinomas (Sonoda *et al*, 1998; Kubokawa *et al*, 2001; Noguchi *et al*, 2001; Takahashi *et al*, 2001). The development of therapeutic tools against RCAS1 would allow us to explore novel targeting therapy in human cancers including uterine endometrial cancer.
